# Double Agglutination Observed in Mycoplasma-Associated Kawasaki Disease: Pathophysiological Insights and Considerations for Treatment Strategy

**DOI:** 10.7759/cureus.99965

**Published:** 2025-12-23

**Authors:** Megumi Akutsu, Daisuke Matsubara, Kazuo Takahashi, Mitsuru Seki, Kazuhiko Kotani

**Affiliations:** 1 Department of Pediatrics, International University of Health and Welfare, Tochigi, JPN; 2 Department of Pediatrics, Jichi Medical University, Tochigi, JPN; 3 Division of Community and Family Medicine, Jichi Medical University, Tochigi, JPN; 4 Department of Laboratory Medicine, International University of Health and Welfare, Tochigi, JPN

**Keywords:** antibody, cold agglutination disease, kawasaki disease, myocoplasma pnemoniae infection, neutrophil agglutination, pathogen-specific treatment, pseudo-leukopenia, thrombosis

## Abstract

We describe a rare pediatric case of *Mycoplasma*-associated Kawasaki disease, accompanied by secondary cold agglutinin disease, characterized by both erythrocyte and neutrophil agglutination. In this case, neutrophil agglutination resolved within one week, while erythrocyte agglutination persisted for four weeks, paralleling the decline in *Mycoplasma* antibody titers and suggesting possible differences in their agglutination thresholds. Erythrocyte agglutination through secondary cold agglutinin disease sometimes facilitates the concomitant diagnosis of *Mycoplasma* infection. On the other hand, neutrophil agglutination is rare, particularly in children, which poses a diagnostic challenge. This case also highlights potential diagnostic considerations and management challenges when Kawasaki disease occurs in the setting of *Mycoplasma* infection. A brief discussion of clinical implications and a review of relevant literature are provided.

## Introduction

*Mycoplasma pneumoniae* is a common respiratory pathogen in children, classified as an atypical bacterium, and can trigger immunological reactions in the host [1]. Secondary cold agglutinin disease (CAD), which causes erythrocyte agglutination, may serve as an indicator of this infection [1,2]. In this context, neutrophil agglutination is a rare phenomenon, particularly in children. Kawasaki disease (KD) is a common vasculitis affecting young children [3], and some cases have been reported to be associated with *M. pneumoniae* infection [4]. Given the serious cardiovascular complications of KD, early diagnosis and treatment are essential. Here, we describe a case of *Mycoplasma*-associated KD with secondary CAD accompanied by both erythrocyte and neutrophil agglutination. A review of relevant literature is also provided.

## Case presentation

A previously healthy six-year-old boy presented with mild respiratory symptoms and a high-grade fever. On illness day five, he was initially diagnosed with acute sinusitis at a local clinic and was treated with oral antibiotics (amoxicillin hydrate), but his symptoms did not resolve. Subsequently, he developed conjunctival injection, a strawberry tongue, an erythematous mouth, and bilateral cervical lymphadenopathy. Upon admission, seven days after disease onset, his vital signs were as follows: body temperature, 38.0 °C; blood pressure, 84/40 mmHg; pulse rate, 123 beats per minute; respiratory rate, 24 breaths per minute; and oxygen saturation, 100% on ambient air. He met four diagnostic criteria for KD according to the Japanese guidelines [3], and incomplete KD was suspected. 

**Figure 1:**
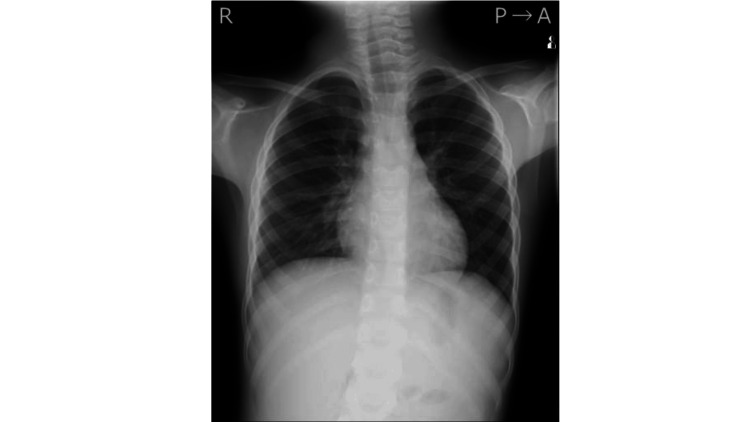
Chest X-ray at admission. A chest X-ray obtained at admission showed no evidence of pneumonia.

**Figure 2:**
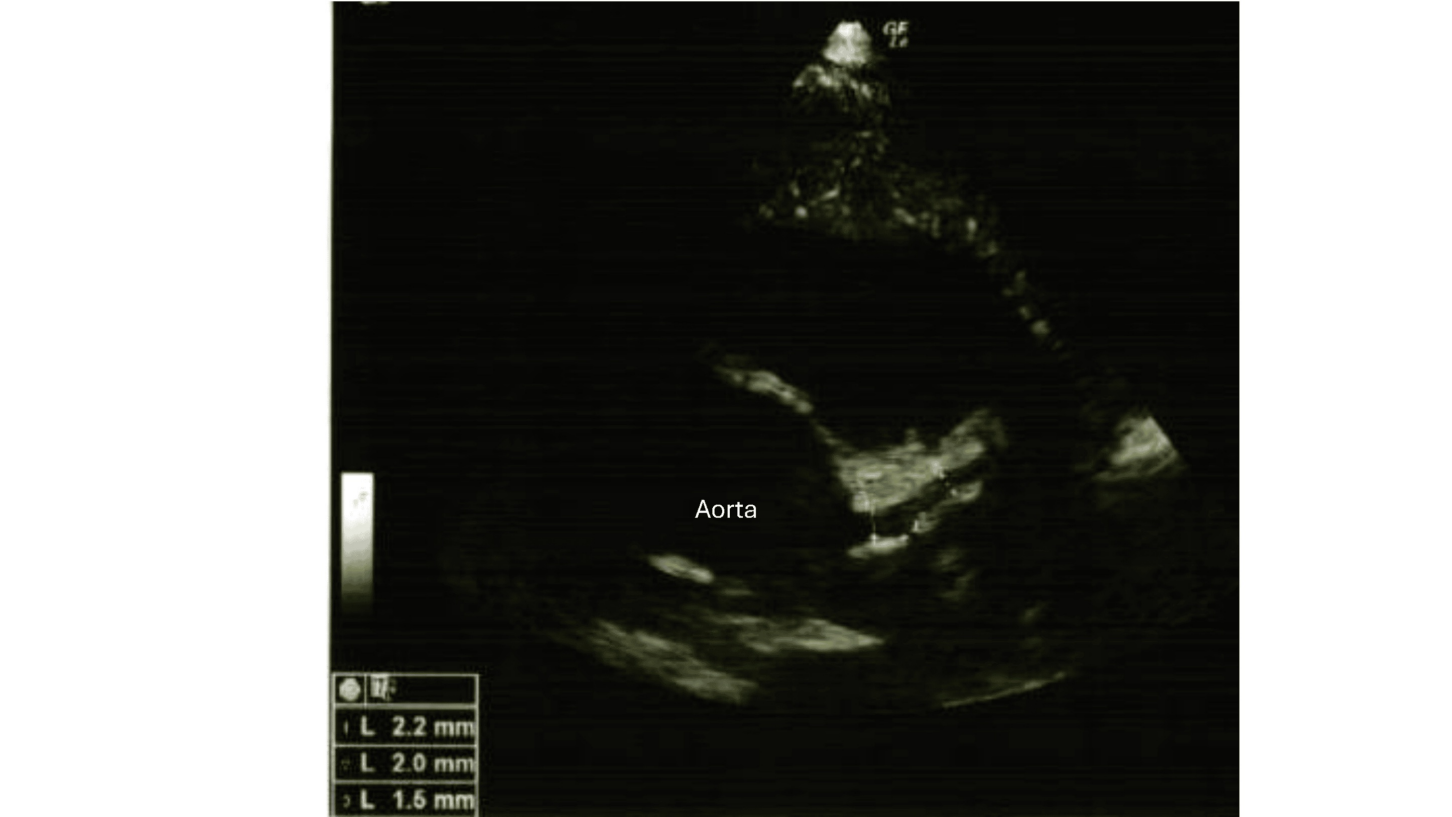
Echocardiography at admission. Echocardiography revealed no cardiac involvement, including coronary arterial lesions.

Blood tests revealed mild inflammation (C-reactive protein, 3.0 mg/dL; white blood cell count, 5,900/μL with 65% neutrophils) and mild hemolysis (total bilirubin, 0.2 mg/dL; lactate dehydrogenase, 427 U/L; hemoglobin, 13.1 g/dL), along with a positive direct Coombs test (Table 1). Although *M. pneumoniae* infection was prevalent in the community at that time, nasal *M. pneumoniae* DNA testing was negative. A chest X-ray obtained at admission showed no evidence of pneumonia (Figure 1). Echocardiography revealed no cardiac involvement, including coronary arterial lesions (Figure 2).

**Table 1:** Laboratory data on admission.

Parameter	Value	Reference Range
White blood cell count (×10^3^/μL)	5.9	3.3–8.6
Neutrophils (%)	65.3	42–74
Hemoglobin (g/dL)	13.1	13.7–16.8
Platelet count (×10^4^/μL)	20.7	15.8–34.8
D-dimer (μg/mL)	0.5	0–1.0
C-reactive protein (mg/dL)	2.95	0–0.14
Albumin (g/dL)	3.2	4.1–5.1
Total bilirubin (g/dL)	0.2	0.4–1.5
Aspartate aminotransferase (U/L)	31	13–30
Alanine aminotransferase (U/L)	18	10–42
Lactate dehydrogenase (U/L)	427	124–222
Creatine phosphokinase (U/L)	37	59–248
Blood urea nitrogen (mg/dL)	7.8	8–20
Creatinine (mg/dL)	0.28	0.65–1.07
High-density lipoprotein cholesterol (mg/dL)	24	38–90
Sodium (mmol/L)	136	138–145
Potassium (mmol/L)	3.5	3.6–4.8
Chloride (mmol/L)	102	101–108
Antinuclear antibody (titer)	<40	0–40
Complement activity (CH50/mL)	25.1	25–48
C3 (mg/dL)	123	73–138
C4 (mg/dL)	10	11–31
Myeloperoxidase anti-neutrophil cytoplasmic antibody (U/mL)	<1.0	0–3.5
Proteinase-3 anti-neutrophil cytoplasmic antibody (U/mL)	1.4	0–3.5

**Figure 3:**
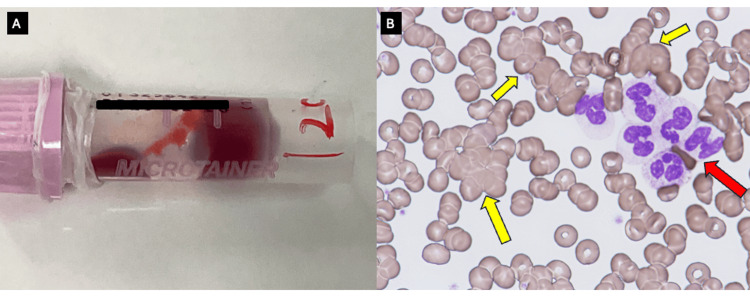
Peripheral blood agglutination and smear findings. (A) Peripheral blood agglutination was observed at room temperature and disappeared after warming to 37 °C, indicating cold agglutination. (B) Both erythrocyte (yellow arrows) and neutrophil (red arrow) agglutination were observed in the peripheral blood smear (May–Giemsa stain, ×400).

Nine days after disease onset, antibiotic therapy with sulbactam/ampicillin (150 mg/kg/day) did not resolve the fever or KD symptoms; therefore, incomplete KD was confirmed. On the same day, peripheral blood agglutination was observed for the first time during the clinical course at room temperature (Figure 3, Panel A) and disappeared after warming to 37 °C, indicating cold agglutination, as noted by a medical technologist. Additionally, both erythrocyte (yellow arrows) and neutrophil (red arrows) agglutination were identified (Figure 3, Panel B), a finding rarely reported in KD. Anti-neutrophil cytoplasmic antibody tests were negative. High titers of cold agglutinins (>1:8192) suggested secondary CAD. Considering the epidemic status of *Mycoplasma* infection and the presence of secondary CAD, concomitant *Mycoplasma* infection was strongly suspected, leading to the diagnosis of *Mycoplasma*-associated KD and secondary CAD with neutrophil aggregation.

The patient was treated with intravenous immunoglobulin (IVIG, 2 g/kg) and oral aspirin (30 mg/kg) for KD, which led to the prompt resolution of both KD and respiratory symptoms. He exhibited no cardiac involvement, including coronary artery lesions and pericarditis, during the course.

**Figure 4:**
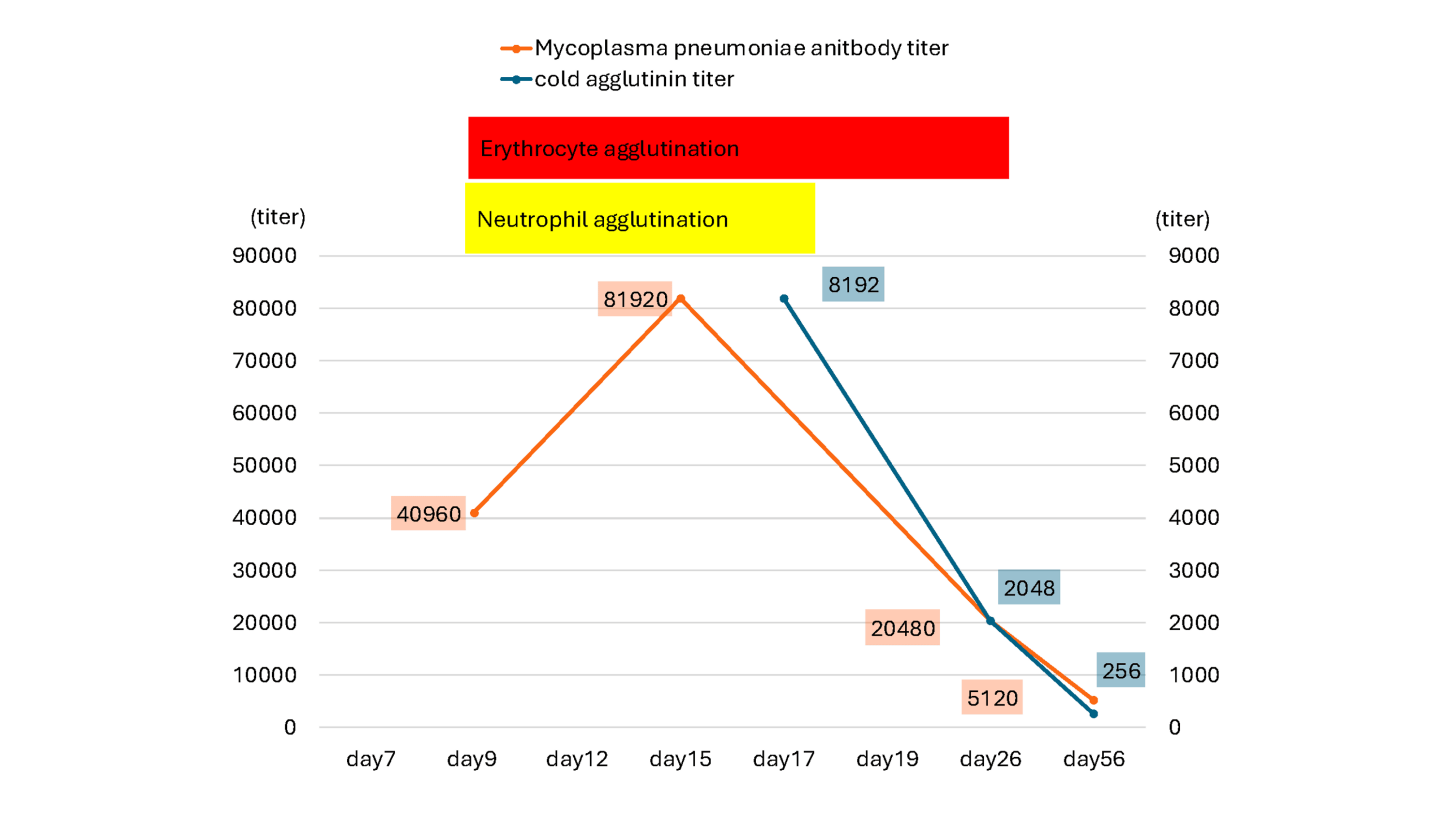
Clinical course of erythrocyte and neutrophil agglutination with Mycoplasma pneumoniae antibody titers. As *M. pneumoniae* antibody titers decreased, neutrophil and erythrocyte agglutination normalized over one and four weeks, respectively. The left Y-axis represents the *M. pneumoniae* antibody titers, and the right Y-axis represents the cold agglutinin titers.

Subsequent testing confirmed elevated *M. pneumoniae* antibody titers (passive agglutination test, 40,960; IgM, 20,480). As *M. pneumoniae* antibody titers decreased, neutrophil and erythrocyte agglutination normalized over one and four weeks, respectively (Figure 4).

## Discussion

We report a pediatric case of *Mycoplasma*-associated KD characterized by both erythrocyte and neutrophil agglutination. Although neutrophil agglutination, a rare phenomenon observed in clinical practice, is generally regarded as having limited clinical significance, the present case is noteworthy for two reasons. First, these agglutinations facilitated the concomitant diagnosis of *Mycoplasma* infection. Second, *Mycoplasma* infection can coexist with KD, warranting special consideration in both diagnosis and management.

In this patient, both erythrocyte and neutrophil agglutinations were identified on day nine after disease onset, which allowed the concomitant diagnosis of *Mycoplasma* infection. Secondary CAD associated with *Mycoplasma* infection, resulting in erythrocyte agglutination, is well recognized [1,2]. Conversely, neutrophil agglutination is a rare occurrence. Leukoagglutination can be classified into two types: (1) ethylenediaminetetraacetic acid (EDTA)-dependent leukoagglutination (the most common) and (2) EDTA-independent cold-induced leukoagglutination [5]. The latter has been described in association with cirrhosis, infections (*M. pneumoniae *infection or infectious mononucleosis), autoimmune diseases, uremia, immunosuppression, and malignancies [5].

Neutrophil agglutination is particularly rare in children, with most cases being associated with *Mycoplasma* infection, as observed in the present case. A review of the literature identified nine pediatric cases, including the present one, of *Mycoplasma* infection accompanied by neutrophil agglutination (Table 2) [6-10]. The ages ranged from 6 to 18 years. Neutrophil agglutination was transient, resolving within two to three weeks in most cases, although one case required five months for normalization. Some patients exhibited additional leukocyte agglutination involving eosinophils, lymphocytes, or monocytes. Almost all cases demonstrated high cold-agglutinin titers (>1:512) and elevated *M. pneumoniae* antibody titers (>1:20,960), findings consistent with the present case. Two patients were also coinfected with herpes simplex virus or Epstein-Barr virus. The present case represents the first report of *Mycoplasma*-associated KD.

**Table 2:** Reported pediatrics cases of neutrophil agglutination associated with Mycoplasma pneumoniae infection. ABPC/SBT, ampicillin/sulbactam; ASA, acetylsalicylic acid; EBV, Epstein–Barr virus; HSV, herpes simplex virus; IVIG, intravenous immunoglobulin; KD, Kawasaki disease; mo, month; MP, *Mycoplasma pneumoniae*; y, years.

Case	Age / Sex	Comorbidity	Treatment	Neutrophil agglutination	Erythrocyte agglutination	Other leukocyte agglutination	Duration of neutrophil agglutination	Duration of erythrocyte agglutination	Cold agglutinin titer	Mycoplasma pneumonia antibody titer	Reference
1	8 y / M	MP	Antibiotic (+)	(+)	Unknown	(-)	Days 13–21	Unknown	>1:1024	>1:40,960	6
2	7 y / M	MP	Antibiotic (+)	(+)	Unknown	(-)	Days 10–14	Unknown	>1:512	>1:40,960	6
3	6 y / F	MP	Antibiotic (+)	(+)	Unknown	(-)	Days 12–14	Unknown	>1:512	>1:10,280	6
4	7 y / M	MP	Antibiotic (+)	(+)	Unknown	Eosinophil	Days 15–32	Unknown	>1:512	>1:40,960	6
5	13 y / F	MP + HSV	Antibiotic (+) (erythromycin)	(+)	Unknown	(-)	Days 15–5 mo	Unknown	unknown	>1:320	7
6	7 y / M	MP + EBV	Antibiotic (+) (amoxicillin → discontinued)	(+)	(+)	(-)	Days 6–11	Days 6–11	>1:4096	Positive	8
7	12 y / F	MP	Antibiotic (+) (azithromycin)	(+)	(+)	Eosinophil, lymphocyte	Unknown	Unknown	<1:64	>1:116	9
8	18 y / F	MP	Antibiotic (+) (levofloxacin)	(+)	(+)	Eosinophil, monocyte	Unknown	Unknown	>1:8192	>1:20,480	10
9 (present case)	6 y / M	MP + KD	Antibiotic (+) (ABPC/SBT) + IVIG + ASA	(+)	(+)	(-)	Days 9–16	Days 9–1 mo	>1:8192	>1:40,960	—

The mechanism of neutrophil agglutination remains undetermined. Erythrocyte agglutination can be explained by anti-I antibodies (cold agglutinins), which bind to the I antigen on the surface of erythrocytes during *M. pneumoniae* infection [11]. Because erythrocyte and neutrophil agglutinations are likely to share a similar underlying mechanism, our close observation that neutrophil and erythrocyte agglutinations normalized over one and four weeks, respectively, following the decline in *Mycoplasma* antibody titers suggests differences in their agglutination thresholds, which may partially explain the varying frequency of each.

KD is a common vasculitis in young children of unknown etiology and is most frequently reported in Japan [3]. To prevent serious cardiovascular complications such as coronary artery lesions, IVIG should be administered as early as possible. Recent meta-analyses have shown that approximately 30% of KD cases coexist with infections, including rhinovirus (19%), adenovirus (10%), and *M. pneumoniae* (10-22%) [4,12]. Among these, *Mycoplasma*-associated KD warrants special attention because its distinct inflammatory mechanisms may contribute to a pathophysiology different from that of non-*Mycoplasma*-associated KD [13]. *M. pneumoniae* infection induces systemic inflammation through multiple mechanisms: (1) direct injury caused by invasion or locally produced inflammatory cytokines; (2) indirect injury mediated by autoimmune reactions and immune complexes; and (3) vascular occlusion resulting from vasculitis or thrombosis [13]. As *Mycoplasma*-associated KD tends to present with prolonged fever after IVIG treatment and increased cardiac involvement compared with non-*Mycoplasma*-associated KD [14], this may be partly explained by immune functional alterations in the host caused by *Mycoplasma* infection [4]. Given the distinctive clinical features of *Mycoplasma*-associated KD, early identification of concomitant *Mycoplasma* infection is essential for an appropriate and timely treatment strategy. Interestingly, in the present case, agglutination observed in the peripheral blood smear provided valuable clues for the early diagnosis of concomitant *Mycoplasma* infection.

Additionally, we propose three clinically important considerations for managing the present case, i.e., *Mycoplasma*-associated KD with secondary CAD and neutrophil agglutination: pseudo-leukopenia, thrombosis, and pathogen-specific treatment. First, neutrophil agglutination can result in pseudo-leukopenia, necessitating careful interpretation of laboratory findings [15], although this was not observed in the present case. Because KD is an inflammatory disorder, the white blood cell count, particularly the neutrophil count, is essential for clinical decision-making and is among the major predictors of IVIG resistance [16]. Therefore, neutrophil agglutination could potentially mislead treatment planning in KD. Second, the presence of CAD may increase the risk of thrombosis [17]. This is particularly important in KD, as coronary arterial lesions represent the most serious complications. Although no cardiac involvement was observed in the present case, physicians should monitor cardiac lesions closely in similar cases. Third, pathogen-specific therapy may be required in cases of *Mycoplasma*-associated KD, as suggested in previous reports [6-10]. The reason why IVIG therapy alone (without antibiotic administration such as macrolides) resulted in prompt resolution of both KD symptoms and respiratory manifestations in the present case remains unclear. Nevertheless, this finding supports that the present case indeed represented true KD, although *Mycoplasma* infection can occasionally mimic KD [18].

## Conclusions

We reported a rare case of KD with secondary CAD accompanied by both erythrocyte and neutrophil agglutination, which facilitated the concomitant diagnosis of *Mycoplasma* infection. Although *M. pneumoniae* DNA testing was initially negative, serological testing later confirmed the diagnosis through elevated *M. pneumoniae* IgM antibody titers. This case is the first to demonstrate a detailed serological course and its association with neutrophil and erythrocyte agglutination, suggesting different thresholds within a shared underlying mechanism. Because *Mycoplasma*-associated KD may require special attention in management, including IVIG treatment with or without antibiotics, this case also provides several clinically important insights for physicians.
